# A Statistical Shape Model of Infrarenal Aortic Necks in Patients With and Without Late Type Ia Endoleak After Endovascular Aneurysm Repair

**DOI:** 10.1177/15266028221149913

**Published:** 2023-01-16

**Authors:** Willemina A. van Veldhuizen, Richte C. L. Schuurmann, Roy Zuidema, Anna C. M. Geraedts, Frank F. A. IJpma, Rogier H. J. Kropman, George A. Antoniou, Marc R. H. M. van Sambeek, Ron Balm, Jelmer M. Wolterink, Jean-Paul P. M. de Vries

**Affiliations:** 1 Department of Surgery, Division of Vascular Surgery, University Medical Center Groningen, Groningen, The Netherlands; 2 Multi-Modality Medical Imaging (M3I) Group, Technical Medical Centre, University of Twente, Enschede, The Netherlands; 3 Department of Surgery, Amsterdam Cardiovascular Sciences, Amsterdam University Medical Centers, University of Amsterdam, Amsterdam, The Netherlands; 4 Department of Surgery, Division of Trauma Surgery, University Medical Center Groningen, Groningen, The Netherlands; 5 Department of Vascular Surgery, St. Antonius Hospital, Nieuwegein, The Netherlands; 6 Department of Vascular and Endovascular Surgery, Manchester University NHS Foundation Trust, Manchester, UK; 7 Division of Cardiovascular Sciences, School of Medical Sciences, Manchester Academic Health Science Centre, The University of Manchester, Manchester, UK; 8 Department of Vascular Surgery, Catharina Hospital, Eindhoven, The Netherlands; 9 Department of Applied Mathematics, Technical Medical Centre, University of Twente, Enschede, The Netherlands

**Keywords:** principal component, abdominal aortic aneurysm, endovascular procedures, endoleak, endovascular aneurysm repair

## Abstract

**Purpose:**

Hostile aortic neck characteristics, including short length, severe suprarenal and infrarenal angulation, conicity, and large diameter, have been associated with increased risk for type Ia endoleak (T1aEL) after endovascular aneurysm repair (EVAR). This study investigates the mid-term discriminative ability of a statistical shape model (SSM) of the infrarenal aortic neck morphology compared with or in combination with conventional measurements in patients who developed T1aEL post-EVAR.

**Materials and Methods:**

The dataset composed of EVAR patients who developed a T1aEL during follow-up and a control group without T1aEL. Principal component (PC) analysis was performed using a parametrization to create an SSM. Three logistic regression models were created. To discriminate between patients with and without T1aEL, sensitivity, specificity, and the area under the receiver operating characteristic (ROC) curve (AUC) were calculated.

**Results:**

In total, 126 patients (84% male) were included. Median follow-up time in T1aEl group and control group was 52 (31, 78.5) and 51 (40, 62.5) months, respectively. Median follow-up time was not statistically different between the groups (p=0.72). A statistically significant difference between the median PC scores of the T1aEL and control groups was found for the first, eighth, and ninth PC. Sensitivity, specificity, and AUC values for the SSM-based versus the conventional measurements–based logistic regression models were 79%, 70%, and 0.82 versus 74%, 73%, and 0.85, respectively. The model of the SSM and conventional measurements combined resulted in sensitivity, specificity, and AUC of 81%, 81%, and 0.92.

**Conclusion:**

An SSM of the infrarenal aortic neck determines its 3-dimensional geometry. The SSM is a potential valuable tool for risk stratification and T1aEL prediction in EVAR. The SSM complements the conventional measurements of the individual preoperative infrarenal aortic neck geometry by increasing the predictive value for late type Ia endoleak after standard EVAR.

**Clinical Impact:**

A statistical shape model (SSM) determines the 3-dimensional geometry of the infrarenal aortic neck. The SSM complements the conventional measurements of the individual pre-operative infrarenal aortic neck geometry by increasing the predictive value for late type Ia endoleaks post-EVAR. The SSM is a potential valuable tool for risk stratification and late T1aEL prediction in EVAR and it is a first step toward implementation of a treatment planning support tool in daily clinical practice.

## Introduction

Hostile infrarenal aortic neck characteristics such as short length (<1 cm), severe suprarenal (>45°) and infrarenal (>60°) angulation, conicity, and large diameter (>30 mm) have been associated with increased risk for a type Ia endoleak (T1aEL) after endovascular aneurysm repair (EVAR).^1–5^ Instructions for use (IFU) of endograft manufacturers include these measurements with predefined cut-off values. These cut-off values are, however, not always based on scientific evidence, and definitions and methods for measuring aortic neck characteristics vary substantially between studies and clinical practices.^6–10^ Also, most studies evaluated the association between a single neck characteristic and post-EVAR complications instead of combining them, which may lead to oversimplification of complex 3-dimensional (3D) geometry.^3,11–14^ The individual characteristics are intertwined in the 3D shape, therefore potentially confounding the outcomes of these studies. There is a need for an alternative approach to describe the full neck geometry in an observer-independent 3D manner and to investigate its association with post-EVAR outcomes.

A statistical shape model (SSM) is a mathematical technique that models the 3D shape variation of an anatomical structure in a population. The SSM is the result of a principal component analysis (PCA) on a parametrization of the aortic neck geometry. The PCA yields linearly independent components to describe shape variation and to capture interactions between geometrical characteristics in a group.^15^ Recently, we introduced an SSM for the aortic neck of infrarenal abdominal aortic aneurysm (AAA) patients who were treated with EVAR and who did not suffer from neck-related complications.^16^ This study investigates the mid-term discriminative ability of an SSM of the infrarenal aortic neck compared with or in combination with conventional measurements in patients who developed T1aEL post-EVAR.

## Methods

### Study Population

A retrospective case-control study was conducted to determine the pre-EVAR shape variation of the infrarenal AAA neck geometry by using statistical shape modeling. A group of patients with a mid-term-to-late T1aEL and an equally large group of patients without T1aEL after EVAR with a comparable duration of post-EVAR follow-up were compared.^17^ Only patients were included with a T1aEL that was diagnosed on a computed tomography angiography (CTA) scan later than the first postoperative CTA scan to avoid endoleaks caused by technical failures during the EVAR procedure.

Inclusion criteria for both groups were a technically successful primary EVAR procedure to treat an infrarenal AAA and the availability of a CTA scan within 6 months pre-EVAR. For the T1aEL group, an additional inclusion criterion was the availability of a CTA scan with a T1aEL that was detected after the first postoperative CTA scan. Exclusion criteria were treatment of a symptomatic or ruptured AAA, adjunct proximal fixation such as additional cuffs or endoanchors, computed tomography (CT) scans with insufficient contrast for assessment in a vascular workstation, and intentional low (>5 mm) positioning of the endoprosthesis relative to the lowest renal artery. As standard of care, all patients received yearly duplex follow-up.

The study was conducted in accordance with the STROBE guidelines and was performed in line with the Declaration of Helsinki.^18^ The study was approved by the local institutional review board (registration no 00287, 2021).

**Figure 1.**
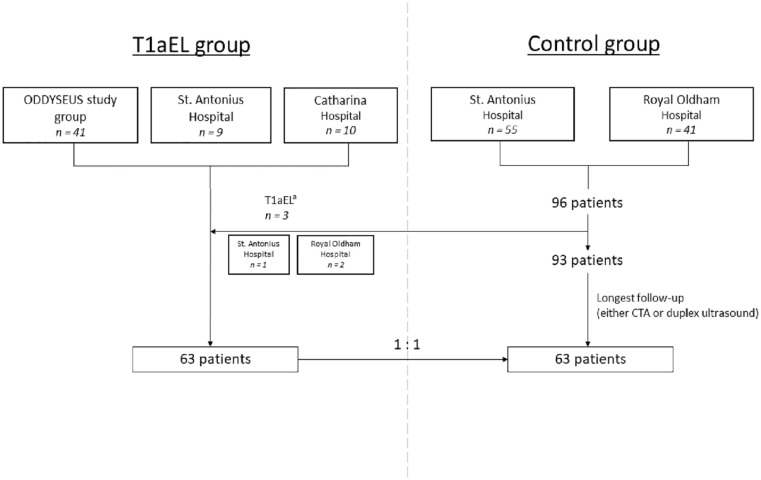
Flowchart of patient inclusion. T1aEL, type Ia endoleak; CTA, computed tomography angiography. ^a^Three patients from the consecutive control cohort were moved to the T1aEL group due to the presence of a T1aEL in the follow-up.

The T1aEL group was composed of patients from 2 previous studies, to include a sufficiently large number of patients with a T1aEL.^19,20^ These patients were treated between 2005 and 2017 in the St. Antonius Hospital, Nieuwegein (n=10), Royal Oldham Hospital, Manchester (n=2), and Catharina Hospital, Eindhoven (n=10). Forty-one patients originated from the ODYSSEUS study group (Figure 1). The patients were treated with Endurant (Medtronic, Santa Rosa, California) (n=39), Talent (Medtronic) (n=13), Zenith (Cook Medical, Bloomington, Indiana) (n=7), and Excluder (W.L. Gore & Associates, Flagstaff, Arizona) (n=4) endografts. The St. Antonius Hospital also participated in the ODYSSEUS study group, so these patients were checked for duplicates.

The control group was composed of consecutive patients who underwent a primary elective EVAR procedure and did not develop a T1aEL during follow-up. This group has also been described in a previous study.^21^ Follow-up scans, either CTA or duplex ultrasound, were evaluated in July 2021 to assess the absence of EVAR-related complications. Patients with the longest duration of CTA or duplex ultrasound follow-up were selected, to match the number of patients in the T1aEL group. These patients were treated with an Endurant II(s) endograft between 2014 and 2017 in the St. Antonius Hospital, Nieuwegein (n=35) and Royal Oldham Hospital, Manchester (n=28).

### Measurements in Vascular Workstation

In the preoperative CTA scan of each patient, the center lumen line and a triangular surface segmentation of the aortic lumen were semi-automatically obtained by experienced researchers following a predefined measurement protocol using a 3mensio Vascular Workstation (version 9.1 SP2, Pie Medical Imaging BV, Maastricht, the Netherlands). On this workstation, conventional measurements, including neck length, neck diameter, suprarenal and infrarenal angulation, calcification, and thrombus, were obtained.^2^ Calcification and thrombus were categorized in 3 different categories according to circumferential presence, namely, absent (<25%), mild (25%–50%), or moderate (>50%).^22^ The 3D markers that serve as input for the parametrization for the SSM were placed at the orifice of the lowest renal artery and at the distal end of the aortic neck, which was defined as a 10% increase in diameter compared with the diameter at the orifice of the lowest renal artery. The center lumen line, segmentation of the aortic lumen, and 3D coordinates were exported to MATLAB 2018a (The MathWorks, Natick, Massachusetts) for further analysis.

### PCA and Statistical Shape Modeling

Dedicated software was designed in MATLAB with a pipeline for preprocessing of the data, alignment of the infrarenal neck segmentations, performing PCA, and creating the SSM. A single SSM was created for all patients, that is, the combined set of patients from the T1aEL and control groups. The data were preprocessed for a PCA of the infrarenal AAA neck by means of a parametrization method, including rigid registration, as described in more detail in a previous publication by our study group.^16^ For each patient, 360 contour points along 10 center lumen line points were created, ensuring anatomical point-to-point correspondence. All infrarenal aortic necks in the dataset were aligned at the lowest renal artery baseline, based on these contour points. The contour points were used as input for a PCA from which the SSM was created. The SSM composed the mean shape of the infrarenal aortic neck by corresponding the paired contour points of all patients in the dataset. All patients in the dataset were compared with this mean shape. As a result, the geometry of each infrarenal AAA neck can be described using scores for all principal components (PCs). The number of PCs is, as default for any SSM, the number of patients – 1, where each PC represents a unique shape variation. Each PC in the SSM describes a percentage of the total variation in the dataset, with a decreasing percentage for each subsequent PC.^15^ Each individual patient obtains a PC score for all the PCs. In this study, the SSM includes all the PCs that together account for 98% of the total variation in the dataset. The shape variance of each individual PC was visualized as a mesh of the mean shape±3 standard deviations (SDs). The quality of the SSM was quantitatively evaluated by calculating the generalization ability, by means of a leave-one-out cross-validation method.^16^

### Statistical Analyses

Potential differences between the T1aEL and control group in age, sex, and total imaging follow-up time were assessed with a Mann-Whitney *U* test. Total imaging follow-up time was defined as the CTA that showed the T1aEL for the T1aEL group and the latest CTA or duplex ultrasound for the control group. Differences in conventional measurements of the preoperative neck length, neck diameter, and supra- and infrarenal angulation were compared between the T1aEL and control groups using the nonparametric Mann-Whitney *U* test, whereas differences in calcification and thrombus categories were assessed using the Fisher exact test. Differences in the distribution of the PC scores of the SSM between the T1aEL and control groups were assessed with a Mann-Whitney U test.

Three logistic regression models were created to discriminate between the T1aEL and control groups. The first model was based on the SSM. The second model was based on the conventional measurements such as neck length, diameter, infra- and suprarenal angulation, calcification and thrombus categories. The third model was based on a combination of the SSM and conventional measurements. Logistic regression analyses were performed using the enter method to obtain a predicted probability for every patient of having a T1aEL. These predicted probabilities were used as input for a receiver operating characteristic (ROC) curve. The area under the ROC curve (AUC) was used as an index for the discriminative value of the SSM, conventional measurements, and the combined SSM and conventional measurements.^23^ Moreover, the predicted probabilities, with a cut-off threshold of 0.5, were used to calculate the sensitivity and specificity of both models for the detection of patients with a T1aEL. Statistical analyses were performed using IBM SPSS statistics, version 23.0 (IBM Corporation, Armonk, New York), with *p* values <0.05 considered significant.

## Results

Both the T1aEL group and control group consisted of 63 patients. Of the 126 patients, 106 patients were male (84%), and the mean age was 74±7 years. Median time to detection of the endoleak on CTA in the T1aEl group was 52 (31, 78.5) months and median CTA follow-up time in the control group was 51 (40, 62.5) months, which was not significantly different (p=0.72). Table 1 shows the baseline characteristics of the groups. Neck length was significantly shorter in the T1aEL group, whereas neck diameter and suprarenal angulation were significantly larger in the T1aEL group. Infrarenal angulation, calcification, and thrombus categories were not significantly different (Table 2). Twenty-five of the 63 patients had a T1aEL that was diagnosed after >5 years of follow-up.

**Table 1.** Baseline Characteristics.

	T1aEL group (n=63)	Control group (n=63)	*p* value
Age, years	75.0 (69.0, 78.0)	75.0 (71.0, 80.0)	0.27
Gender, male	52 (83)	54 (86)	0.62
Time between pre-EVAR scan and EVAR procedure, months	1.0 (1.0, 2.0)	1.0 (0.0, 1.0)	0.22
Total follow-up (CTA or duplex^a^), months	52.0 (31.0, 78.5)	51.0 (40.0, 62.5)	0.72

Data are presented as median (Q1, Q3) for continuous data or n (%) for categorical data.

Abbreviations: CTA, computed tomography angiography; EVAR, endovascular aneurysm repair; T1aEL, type Ia endoleak.

a Duplex follow-up only applies to the control group.

**Table 2.** Statistical Analyses of Conventional Measurements.

	T1aEL group (n=63)	Control group (n=63)	*p* value
Neck length, mm	13.0 (6.0, 24.6)	26.0 (16.0, 37.0)	**0.00**
Neck diameter, mm	26.1 (23.1, 27.6)	24.3 (22.6, 25.3)	**0.00**
Suprarenal angulation, degrees	36.0 (24.5, 59.0)	30.0 (19.0, 42.0)	**0.02**
Infrarenal angulation, degrees	52.0 (40.0, 69.5)	47.0 (36.5, 61.0)	0.30
Calcification, category	0.0 (0.0–0.0)	1.0 (0.0–1.0)	0.33
Thrombus, category	0.0 (0.0–1.0)	1.0 (0.0–2.0)	0.91

Data are presented as median (Q1, Q3). All statistically significant *p* values (< 0.05) are highlighted in bold.

Abbreviation: T1aEL, type Ia endoleak.

In total, 125 PCs (number of patients – 1) were calculated by PCA. The first 9 PCs together described 98% of the total variation in the dataset. The SSM with these 9 PCs was able to reconstruct a given new patient-specific shape with an accuracy of 3.4 (95% confidence interval [CI]=3.1–3.8) mm.

**Figure 2.**
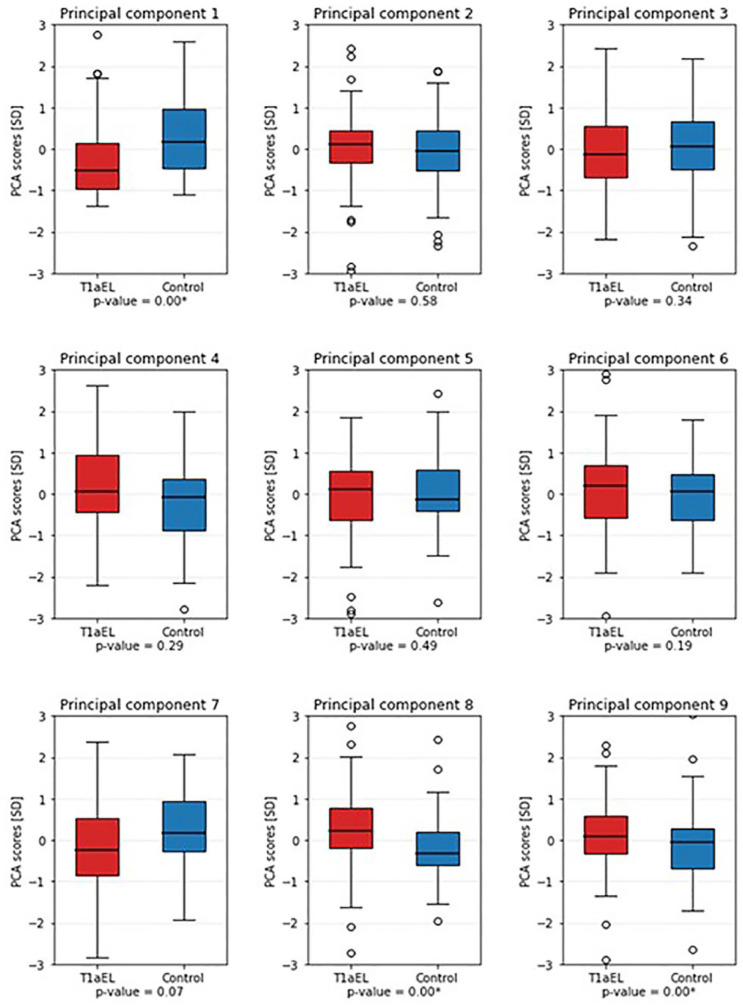
Boxplots of the first 9 principal components, of the T1aEL group (red) versus the control group (blue). The first, eighth, and ninth PCs were significantly different between the groups. PCA, principal component analysis; T1aEL, type Ia endoleak.

In Figure 2, boxplots of these 9 PCs are shown. A statistically significant difference between the median PC scores of the T1aEL and control groups was found for PCs 1, 8, and 9.

**Figure 3.**
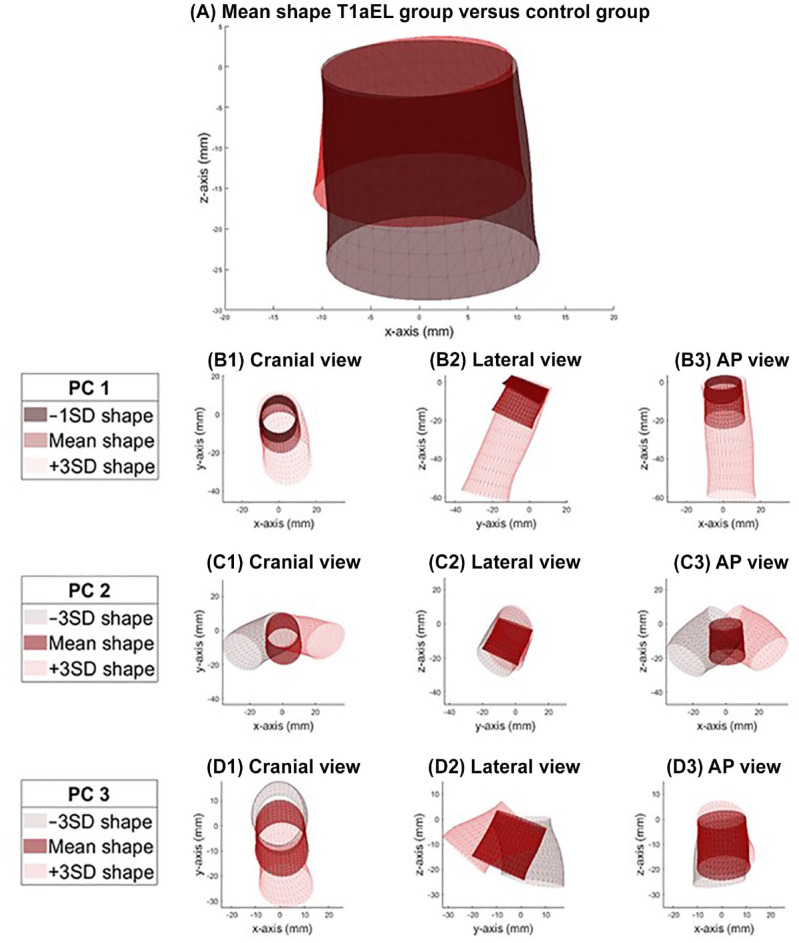
(A) Anteroposterior view of the mean shape of the T1aEL group (red) versus the control group (brown). (B) PC 1 describes 54% of the total shape variation, mainly variation in neck length. (C) PC 2 describes 27% of the total shape variation, mainly left and right deflection. (D) PC 3 which describes 9% of the total shape variation, mainly anterior and posterior deflection. T1aEL, type Ia endoleak; PC, principal component.

Figure 3A shows the mean shape of the T1aEL group (red) and the mean shape of the control group (brown). Figure 3B–D shows the mean shape, –3SD shape variation, and +3SD shape variation for PCs 1, 2, and 3. The individual PCs 1, 2, and 3 describe 54%, 27%, and 9% of the total shape variation, respectively. Note that these components do not directly correspond to a conventional measurement. However, Figure 3B–D suggests that PC 1 mainly describes variation in neck length, PC 2 mainly describes deflection of the aortic neck to the left and right axes, and PC 3 mainly describes anterior and posterior deflection.

**Figure 4.**
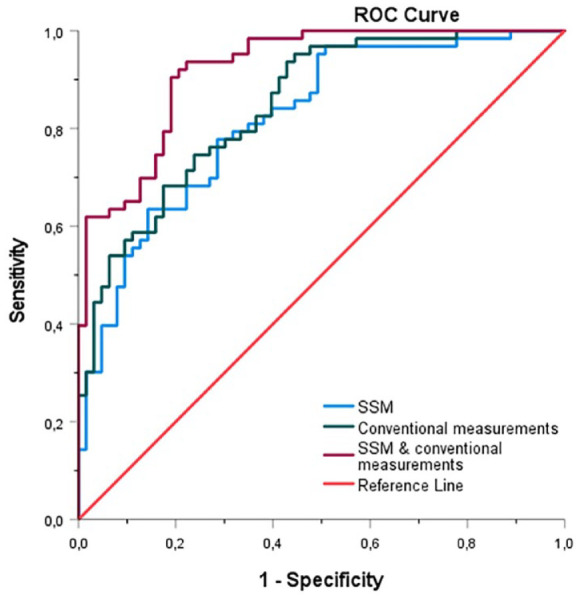
ROC curve of the SSM, including the first 9 principal components (blue), the ROC curve of the conventional measurements, including neck length, diameter, supra- and infrarenal angulation, calcification and thrombus categories (green) and the ROC curve of the combination of the SSM and conventional measurements (purple). ROC, receiver operating characteristic; SSM, statistical shape model.

For the SSM-based logistic regression model, including the first 9 PCs, the sensitivity, specificity, and AUC values were 79%, 70%, and 0.82, respectively. For the conventional measurements–based logistic regression model, the sensitivity, specificity, and AUC values were 74%, 73%, and 0.85, respectively. The third logistic regression model, based on the combination of the SSM and conventional measurements, resulted in sensitivity, specificity, and AUC values of 81%, 81%, and 0.92, respectively. The ROC curves of the 3 models are displayed in Figure 4.

## Discussion

This study describes an SSM that determines the infrarenal aortic neck geometry. By using this semi-automated SSM, complex aortic neck shape variations could be obtained, which could not be obtained from conventional neck characteristics. The 2 logistic regression models that were created, the first based on the SSM and the second model based on conventional neck measurements, resulted in comparable values for sensitivity, specificity, and AUC to discriminate between the control and TIaEL groups. A combination of the SSM and conventional measurement model resulted in the highest sensitivity, specificity, and AUC values, which suggests that the SSM is taking into account more information from the aortic neck geometry than conventional measurements could provide.

The SSM considers the infrarenal abdominal aortic neck as a true 3D geometrical shape, instead of separate single neck characteristics. The conventional measurements included in this study were limited to neck length, neck diameter, infra- and suprarenal angulation, and calcification and thrombus categories, as these measurements are most commonly evaluated in the current literature and are included in international guidelines.^6,24–26^ It should be realized that the conventional measurements in this study were performed in a controlled setting, where experienced researchers performed the measurements according to a predefined protocol, which may differ from daily practice. An SSM can therefore reduce the measurement variability. However, measuring neck characteristics is still of great value for surgical preparation and sizing and planning of an endograft. Therefore, implementation of the SSM in clinical practice should be regarded not as a replacement, but rather as a valuable addition to the conventional measurements.

To create an SSM, the center lumen line, a semi-automated segmentation of the aortic lumen, and coordinates of the lowest renal artery and distal end of the neck (which were manually positioned) are needed. Neck length, a prerequisite for parametrization in the SSM, is the only subjective conventional measurement the SSM depends on, in contrast to the conventional measurements, which are all subjective to observer variability.^8–10^ In the current study, the 3mensio Vascular Workstation was used for the measurements and input for the SSM. The required input for the SSM could, however, also be obtained in any other vascular workstation with a centerline reconstruction. The SSM is based on manual measurements of the aortic neck, which could be of influence on the SSM outcomes. However, the intraobserver and interobserver variability of neck length measurements showed to be low, with a median difference of 0.6 mm (intraclass correlation coefficient=0.994).

The neck length measurement, which is also input for the SSM, may be further automated in the future by automatic segmentation of the infrarenal aortic neck using artificial intelligence (AI)–based solutions.^27,28^ The SSM and future AI can be further developed and improved with a larger dataset of AAA patients. Additional parameters such as apposition of the endograft with the aortic wall, derived from the postoperative CT scan, could also be included in a future version of the SSM. By doing so, the SSM might aid vascular surgeons in clinical decision-making (eg, EVAR vs open surgical repair or fenestrated/branched repair), by calculating a patient-tailored risk for T1aEL, based on their actual preoperative 3D anatomy.

The use of an SSM as a discriminative tool in the vascular field is relatively new and unexplored territory. Previous studies created an SSM of the thoracic aorta to explore the aortic geometry and to associate shape with ventricular function, wall shear stress, or rupture risk.^23,29,30^ It can be expected that the role of such models during clinical decision-making will increase in the future as the models become more robust.

This study considered the anatomical variation of patients who had a late T1aEL. Future research could also look at perioperative anatomical variation.

Besides the relatively small patient groups, this study has some other limitations. A potential bias might have been introduced in the data collection, as patients in the T1aEL group were treated earlier than the patients in the control group. Moreover, the control group was treated with the Endurant endograft, whereas a variety of endografts was used in the T1aEL group, including the Talent endograft. This type of endograft did not have external fixation, which made it more prone to migration compared with the present-day endografts. Furthermore, a limitation of this study is that the results were not stratified according to endograft type.

Besides challenging preoperative neck anatomy, a late T1aEL can also originate from other causes such as progressive disease, an inadequate seal between endograft and aortic wall due to endograft migration, or as a result of aneurysm growth due to another endoleak, for example, type II.^31–36^ Type II endoleak can lead to sac pressurization and growth of the aneurysm, including progression of the disease of the infrarenal neck, therefore causing a type Ia endoleak later on. Unfortunately, information about type II endoleak was not complete in the retrospective dataset, and it could not be verified for all patients.

Another limitation was that in the control group, CTA and duplex ultrasound follow-up were used to verify the absence of a T1aEL. The sensitivity of detecting an endoleak may be lower in duplex ultrasound, so the presence of an endoleak might potentially be missed.^36^ This model is a first step toward implementation of a treatment planning support tool in daily clinical practice.

## Conclusion

An SSM of the infrarenal aortic neck determines its 3D geometry. The SSM is a potential valuable tool for risk stratification and late T1aEL prediction in EVAR. The SSM complements the conventional measurements of the individual preoperative infrarenal aortic neck geometry by increasing the predictive value for late T1aEL after standard EVAR.
